# *In Vitro* Anti-Inflammatory and Anticancer Evaluation of *Mentha spicata* L. and *Matricaria chamomilla* L. Essential Oils

**DOI:** 10.1021/acsomega.3c01501

**Published:** 2023-05-06

**Authors:** Sevde Nur Biltekin, Ayşe Esra Karadağ, Fatih Demirci, Betül Demirci

**Affiliations:** † Department of Pharmaceutical Microbiology, School of Pharmacy, Istanbul Medipol University, 34815 Istanbul, Türkiye; ‡ Department of Molecular Biology and Genetics, Institute of Graduate Studies in Sciences, Istanbul University, 34452 Istanbul, Türkiye; § Department of Pharmacognosy, İstanbul Medipol University, Faculty of Pharmacy, 34815 İstanbul, Türkiye; ∥ Department of Pharmacognosy, Faculty of Pharmacy, Anadolu University, 26470 Eskişehir, Türkiye; ⊥ Faculty of Pharmacy, Eastern Mediterranean University, 99450 Famagusta, Northern Cyprus, Türkiye

## Abstract

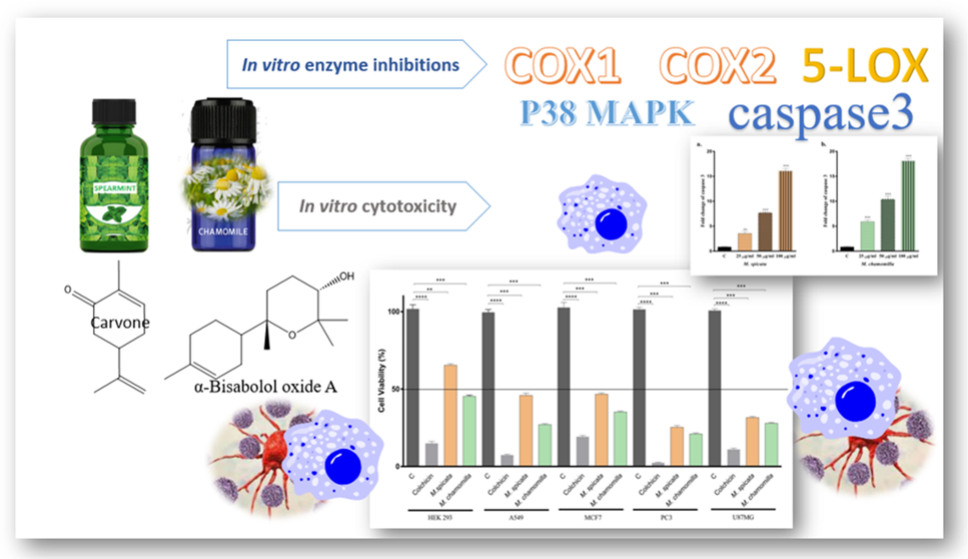

Spearmint, *Mentha spicata* L., and the German chamomile, *Matricaria chamomilla* L., preparations are used against inflammatory conditions traditionally and in modern medicinal applications. This present study aimed to evaluate pharma-grade essential oils for their *in vitro* anti-inflammatory and anticancer effects using COX-1, COX-2, and 5-LOX enzyme assays, as well as their apoptosis potential through the caspase pathway. In addition, the (3-[4,5-dimethylthiazol-2-yl]-2,5 diphenyl tetrazolium bromide) (MTT) assay was applied to evaluate the *in vitro* cytotoxic effects using HEK293/A549, MCF7, and PC3 cell lines. Major components of *M. spicata* essential oil were confirmed both by gas chromatography (GC)-flame ionization detector (FID) and GC/mass spectrometry (MS) as 72.8% carvone, 12.6% limonene, 2.2% 1,8-cineole, 1.3% myrcene, and 1% *trans*-dihydrocarvone. The major components of *M. chamomilla* essential oil were also confirmed as 47.9% α-bisabolol oxide A, 16.8% α-bisabolol, 13.8%, (*Z*)-β-farnesene, 5.8% α-bisabolol oxide, and 4.7% α-bisabolene oxide A. The IC_50_ values for *M. chamomilla* essential oil on A549, MCF7, PC3, and HEK293 cells were calculated as 208.54 ± 1.39, 315.44 ± 1.17, 197.52 ± 0.98, and 638.79 ± 1.15 μg/mL, respectively, whereas the IC_50_ values for *M. spicata* essential oil on A549, MCF7, and PC3 cells were 672.13 ± 2.57, 708.27 ± 2.05, and 206.49 ± 1.48 μg/mL, respectively. For *M. spicata* essential oil, no cytotoxic effects on healthy HEK293 cells were observed at the tested concentrations. The essential oils increased the apoptotic activity, where all results were statistically significant. According to the anti-inflammatory evaluation, both *M. chamomilla* and *M. spicata* oils showed selective COX-2 inhibitions, where the SI values were calculated as 0.30 and 0.67, respectively. Overall, both *M. spicata* and *M. chamomilla* essential oils showed selective inhibition on the COX-2 enzyme and apoptosis against the selected cancer cell lines for the first time, to the best of our knowledge, with this specific dual mode of action. The initial results encourage further detailed *in vivo* experimental evaluations.

## 1 Introduction

Herbal drugs and their preparations still constitute an important resource and natural dispensary against a wide range of ailments. It is well known that the study materials of this present work, the German chamomile *Matricaria chamomilla* L. (syn: *Matricaria recutita* L.), is a member of the Asteraceae, whereas the Spearmint *Mentha spicata* L. belongs to the Lamiaceae systematically. *M. spicata* preparations are traditionally used for their anti-inflammatory effect among others.^1^ In previous studies, *M. spicata* essential oil showed an *in vivo* anti-edema effect.^2,3^*M. chamomilla* and its preparations are recognized for their traditional uses as well as for their anti-inflammatory experimental potential.^4,5^ The anti-inflammatory effects for *M. chamomilla* essential oil were also demonstrated by different *in vivo* and *in vitro* studies.^6^

Cyclooxygenase 2 (COX-2) is a known target for the relief of pain and for the treatment of inflammation and is also known for its contribution to the modulation of multiple procarcinogenic effects.^7,8^ COX-2 is a membrane-bound enzyme, which is also overexpressed in many types of cancer, promoting carcinogenesis and increasing cancer cell resistance to chemo- as well as radiotherapy.^9,10^ The stability in COX-2 mRNA is regulated by p38 mitogen-activated protein kinase.^11^ MAPK, target serine threonine residues in proteins, plays an important role in several biological processes, such as cell proliferation, differentiation, apoptosis, inflammation, and response to environmental stress.^12^ The COX-2 enzyme, which is also responsible for the phosphorylation of proteins, makes cancer cells resistant to chemotheraphy and radiotherapy, and P38/COX-2 is known to be involved in cancer cell resistance to apoptosis.^13^ A possible interaction between MAPK activity, EGFR/p38, and COX-2 promotes angiogenesis in cancer cells is documented.^14^ The epidermal growth factor receptor (EGFR) is a member of the tyrosine kinase superfamily of receptors, where dual positive feedback with COX-2 is evident, which may enhance the carcinogenesis process.^14^ The overexpression of EGFR independent from COX-2 was reported to play a role in the development of many human cancers, including lung, colorectal, breast, prostate, ovarian, and brain cancers.^15^

To the best of our knowledge, this is the first extensive comparative *in vitro* COX-1, COX-2, and 5-LOX inhibitory evaluation of *M. spicata* and *M. chamomilla* essential oils. The *in vitro* cytotoxic effects on different cancer cell lines, such as A549, MCF7, PC3, and healthy HEK293 control cells, were screened along with their effects on targeted apoptosis using the caspase-3, EGFR, and p38 MAPK enzyme assays.

## 2 Results and Discussion

### 2.1 Phytochemical Analyses

The study material of this present work, *M. spicata* essential oil, was confirmed by 72.8% carvone, 12.6% limonene, 2.2% 1,8-cineole, 1.3% myrcene, and 1% *trans*-dihydrocarvone major constituents, whereas the major components for *M. chamomilla* essential oil were identified as 47.9% α-bisabolol oxide A, 16.8% α-bisabolol, 13.8%, (*Z*)-β-farnesene, 5.8% α-bisabolol oxide B, and 4.7% α-bisabolene oxide A, where the detailed GC-FID and GC/MS compositions can be followed in Table 1. While monoterpenes were found more in *M. spicata* essential oil, *M. chamomilla* essential oil was found rich in sesquiterpenes (Table 1).

**Table 1:** Chemical Compositions of *M. chamomilla* and *M. spicata* Essential Oils

RRI^a^	compound	Mc (%)	Ms (%)	IM
1032	α-pinene		0.5	*t*_R_, MS
1076	camphene		0.1	*t*_R_, MS
1118	β-pinene		0.5	*t*_R_, MS
1132	sabinene		0.3	*t*_R_, MS
1174	myrcene		1.3	*t*_R_, MS
1188	α-terpinene		0.2	*t*_R_, MS
1203	limonene		12.6	*t*_R_, MS
1213	1,8-cineole		2.2	*t*_R_, MS
1246	(*Z*)-β-ocimene		0.1	MS
1255	γ-terpinene		0.3	*t*_R_, MS
1266	(*E*)-β-ocimene		tr	MS
1280	*p*-cymene		0.3	*t*_R_, MS
1290	terpiolene		0.1	*t*_R_, MS
1345	3-octyl acetate		0.1	MS
1398	3-octanol		0.4	MS
1443	*trans*-sabinene hydrate		0.5	MS
1475	menthone		0.1	*t*_R_, MS
1528	α-bourbonene		0.1	MS
1535	β-bourbonene		0.9	MS
1574	menthyl acetate		0.1	*t*_R_, MS
1579	terpinen-4-ol		0.6	*t*_R_, MS
1589	β-ylangene		0.1	MS
1612	β-caryophyllene		0.7	*t*_R_, MS
1624	*trans*-dihydrocarvone		1.0	MS
1638	menthol		0.1	*t*_R_, MS
1667	(*Z*)-β-farnesene	13.8		MS
1704	γ-muurolene	0.1		MS
1706	α-terpineol		0.7	*t*_R_, MS
1726	germacrene D	1.1		MS
1732	neo-dihydrocarveol		0.5	MS
1751	carvone		72.8	*t*_R_, MS
1755	bicyclogermacrene	0.7		MS
1758	(*E,E*)-α-farnesene	0.5		MS
1773	δ-cadinene	0.2		MS
1845	*trans*-carveol		0.3	*t*_R_, MS
1882	*cis-*carveol		0.3	*t*_R_, MS
1969	*cis*-jasmone		0.2	MS
2098	globulol	tr		MS
2104	viridiflorol		0.2	MS
2144	spathulenol	0.5		MS
2156	α-bisabolol oxide B	5.8		MS
2187	T-cadinol	0.7		MS
2200	bisabolene oxide A	4.7		MS
2232	α-bisabolol	16.8		*t*_R_, MS
2298	decanoic acid	0.4		*t*_R_, MS
2430	chamazulene	3.5		*t*_R_, MS
2438	α-bisabolol oxide A	47.9		MS
2500	pentacosane	0.7		*t*_R_, MS
2600	hexacosane	0.1		*t*_R_, MS
	monoterpene hydrocarbons		16.2	
	oxygenated monoterpenes		79.3	
	sesquiterpene hydrocarbons	16.4	1.8	
	oxygenated sesquiterpenes	76.4	0.2	
	fatty acids	0.4		
	others	4.3	0.7	
	total	97.5	98.2	

a RRI: Relative retention indices calculated against *n*-alkanes; % calculated from FID data; t: trace (<0.1%); IM: identification method; *t*_R_, identification based on the retention times of genuine compounds on the HP Innowax column; MS, identified on the basis of computer matching of the mass spectra with those of the Wiley and MassFinder libraries and comparison with literature data; Mc: *M. chamomilla* essential oil; *M. spicata* essential oil.

It is well known that *M. chamomilla* has different chemotypes.^16^ In a previous study with 6 different *M. chamomilla* essential oils, (*Z*)-β-farnesene was found in only one of the 6 different samples studied, while (*E*)-β-farnesene was detected at relatively high rates in all analyzed samples. The reported ratio of this study of α-bisabolol oxide B and α-bisabolol oxide A was consistent and comparable with the previous reports.^16^ The major components of *M. spicata* essential oil previously reported by our group show similar and consistent results with the sample tested in this study.^17^ The major oil components of another previous *M. spicata* essential oil report^18^ were also comparable to this present study. The essential oils were also in compliance with the European Pharmacopoeia monographs.

### 2.2 Cytotoxic Activity

**Figure 1:**
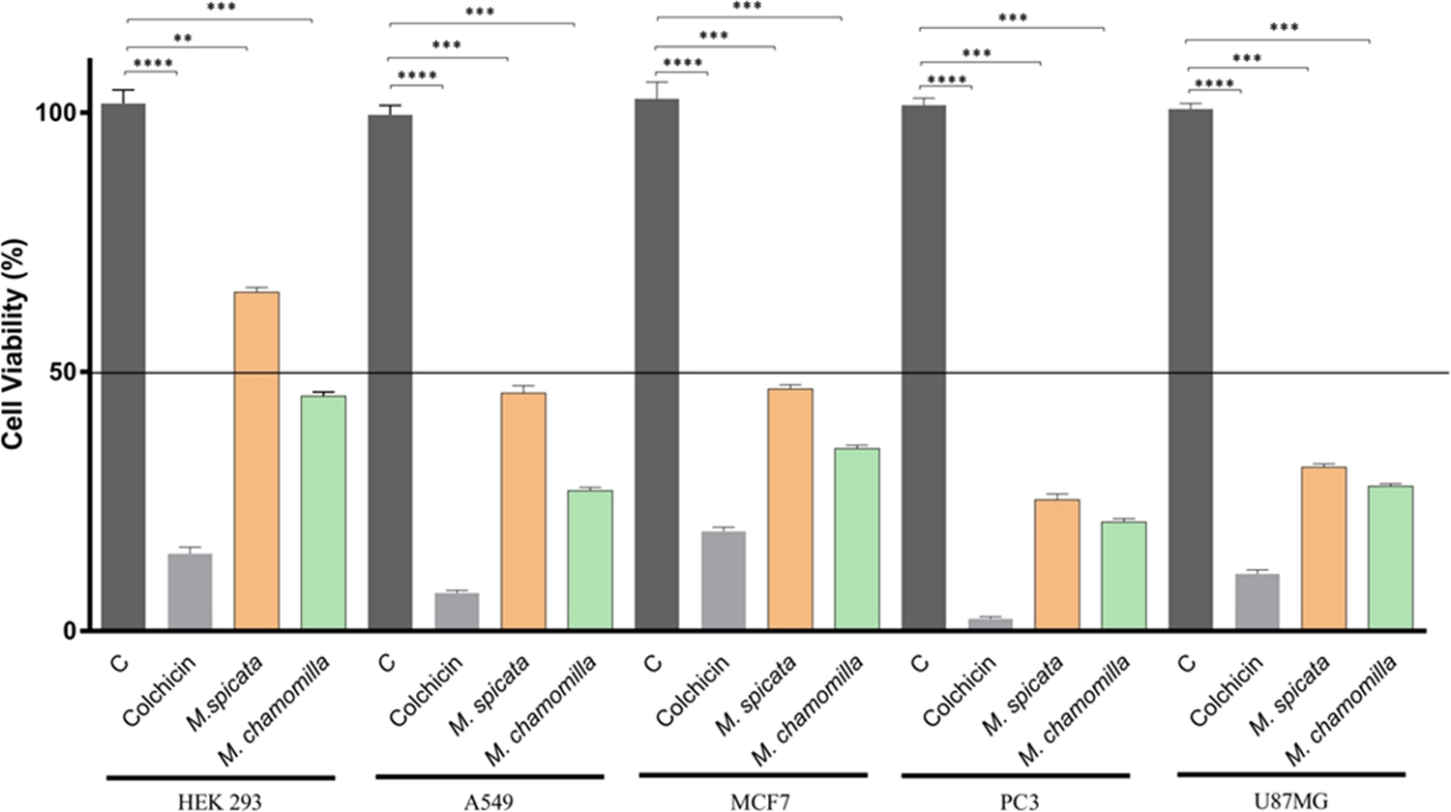
Effects of *M. spicata* (2000 μg/mL) and *M. chamomilla* (2000 μg/mL) essential oils on the viability on cell types for 24 h (mean ± SD, *n* = 5, ***P < 0.001, ****P < 0.0001). Vertical bars indicate standard deviation values, determined by one-way ANOVA, using Dunnet’s multiple comparison test (C: Negative control).

The *in vitro* cytotoxic activity of *M. spicata* and *M. chamomilla* essential oils on the HEK293, A549, MCF7, PC3, and U87MG cells was examined in different concentrations in the range of 0.1–2000 μg/mL. Cytotoxic activity data showed decreased viability in the A549, MCF7, U87MG, and PC3 cell lines after 24 h of incubation of *M. spicata* in a concentration-dependent manner as illustrated in Figure 1. After 24 h of *M. spicata* oil exposure, no cytotoxic effects were observed on healthy HEK293 cells. Moreover, the cell viabilities were about 65% at a concentration of 2000 μg/mL *M. spicata* oil, whereas the IC_50_ values of *M. spicata* essential oil on A549, MCF7, PC3, and U87MG cells were observed as 672.13 ± 2.57, 708.27 ± 2.05, 206.49 ± 1.48, and 218.86 ± 2.11 μg/mL, respectively, as listed in Table 2. The findings of this present study showed that the pharmaceutical-grade *M. spicata* essential oil exerted significant cytotoxic effects on various cancer cell lines and showed selective effects when compared with healthy cells. The cytotoxic activity of *M. spicata* extracts was reported on different cell lines tested in previous studies.^19^ Another previous study, where *M. spicata* essential oil nanofibers were evaluated against A-375 melanoma cells, showed an effective concentration at 1 mg/mL.^18^ The pharmaceutical quality *M. spicata* essential oil was evaluated for the first time to the best of our knowledge.

**Table 2:** IC_50_ (Mean ± SD) Values of the Tested Essential Oils on Cancer Cells (μg/mL)^a^

	IC_50_ (μg/mL)				
sample	A549	PC3	MCF7	U87MG	HEK293
*M. spicata*	672.13 ± 2.57	206.49 ± 1.48	708.27 ± 2.05	218.86 ± 2.11	ND
*M. chamomilla*	208.54 ± 1.39	197.52 ± 0.98	315.44 ± 1.17	172.65 ± 1.93	638.79 ± 1.15
colchicine (std)	9.28 ± 1.01	0.98 ± 0.99	52.11 ± 0.85	18.11 ± 1.12	24.53 ± 1.02

a ND: not detected.

The *M. chamomilla* essential oil of the present study showed relatively more cytotoxicity on the tested four cancer cell lines compared to *M. spicata* oil. The IC_50_ values for *M. chamomilla* essential oil on A549, MCF7, PC3, and U87MG cells were observed as 208.54 ± 1.39, 315.44 ± 1.17, 197.52 ± 0.98, and 172.65 ± 1.93 μg/mL, respectively, as listed in Table 2. The IC_50_ value of the tested *M. chamomilla* essential oil on the HEK293 normal cell line was calculated as 638.79 ± 1.15 μg/mL. However, the selectivity of the tested essential oils showed more significant results when compared to the positive controls. It is well known that *M. chamomilla* preparations are used due to their analgesic, antimicrobial, and anti-inflammatory properties. *M. chamomilla* tea preparations were reported for their cytotoxic effects in previous studies.^20^ The cytotoxic effect of *M. chamomilla* essential oil against MCF-7 cells was also investigated in another previous study, however, was not effective at 1 mg/mL concentration.^21^ Interestingly, the cytotoxic effect of essential oil on MCF-7 cells in this present study was observed, which may be associated with the Pharmacopoeia quality of the oil. The relatively high selectivity of *M. spicata* essential oil toward cancer cells can be considered as an outcome of this present study. *M. chamomilla* essential oil also showed selective cytotoxicity on cancer cells, although not as much as *M. spicata* essential oil when compared. As a result of cytotoxicity targeting, only cancer cells were affected selectively, without damaging healthy cells, which stimulated the work to focus on further anticancer pathways.

In this present study, the toxicities of colchicine and essential oils were used as positive controls in the normal healthy control group, where cancer cell lines were evaluated comparatively. The IC_50_ values were calculated by % inhibition of essential oils and colchicine at different concentrations as listed in Table 2, followed by Table 3 with corresponding selective indices, respectively. According to Table 2 and Table 3 data, although the effect of colchicine in the MCF7 cell line was 6-fold more than the effect of the essential oil, its selectivity was relatively lower due to the high toxicity observed in the HEK293 cell line. For this reason, the safe application may be rather narrow. At the same time, the essential oils used in the study are known to be safe in terms of toxicity and are commonly used both in phyto- and aromatherapy applications and also in various combinations. As it is well known, colchicine is used for prevention and treatment of gout-associated pain, however, the safe dose range, due to the narrow therapeutic index, needs still detailed evaluations and research.

**Table 3:** Selectivity Index (SI) on Cell Toxicity of the Tested Essential Oils

	SI			
sample	IC_50(HEK293)_/IC_50(A549)_	IC_50(HEK293)_/IC_50(PC3)_	IC_50(HEK293)_/IC_50(MCF7)_	IC_50(HEK293)_/IC_50(U87MG)_
*M. spicata*	ND	ND	ND	ND
*M. chamomilla*	3.06	3.23	2.02	3.69
colchicine (std)	2.64	25.03	0.47	1.35

Findings in the present work suggested that both essential oils showed selective cytotoxicity on the tested cancer cells. The most effective oils were on U87MG and PC3 cells, compared to the standard colchicine as illustrated in Table 3. The selective toxicity compared to healthy cells can be justified and classified as promising data for further evaluation. The IC_50_ value of each cancer cell line was calculated by dividing the IC_50_ value of the healthy cell line HEK293.

### 2.3 COX-1, COX-2, and 5-LOX Enzyme Inhibitions

The potential anti-inflammatory effects of both essential oils were calculated by using the *in vitro* COX and LOX enzyme activities. The IC_50_ values were calculated by evaluating the COX-1 and COX-2 enzyme inhibition rates of essential oils at different concentrations. Celecoxib, SC-560, and Dup-697 were used in the COX assays as positive controls. The IC_50_ values of *M. spicata* essential oil on COX-1 and COX-2 enzymes were 21.17 ± 0.85 and 14.28 ± 0.93 μg/mL, respectively. The IC_50_ values of COX-1 and COX-2 for *M. chamomilla* were 39.05 ± 1.02 and 11.83 ± 0.87 μg/mL, respectively, as listed in Table 4. Comparing the results, essential oils showed selective inhibition on COX-2, with *M. spicata* oil having the SI 0.67, and *M. chamomilla* oil with SI 0.30, respectively, as shown in Table 4. In a previous study, extracts of *M. chamomilla* flowers were also investigated for COX enzyme inhibition due to their traditional use in the treatment of anti-inflammatory diseases.^22,23^ The effects of *M. chamomilla* essential oil on some inflammatory cytokines, including COX-2, were reported in previous studies.^24^ The anti-inflammatory effect of *M. spicata* essential oil was investigated *in vivo*, and successful results were obtained previously.^1^ However, to the best of our knowledge, this is the first study on COX enzyme inhibitions by *M. spicata* essential oil. The COX enzyme inhibition results obtained from this study confirm the ethnobotanical anti-inflammatory use and explain the selectivity in the cytotoxic effect for both essential oils. It is well known that COX is a popular target enzyme for the treatment of pain relief and inflammation; furthermore, in recent reports, the enzyme was associated with several cancer types.^9^ It was reported that the overexpression of the COX-2 enzyme promotes carcinogenesis, increases cancer recurrence rate, and decreases survival. COX-2 also plays a role in suppressing apoptosis in cancer cells, cancer migration, metastasis, and invasion.^25^

**Table 4:** Anti-inflammatory Enzyme Inhibitions by Mint and Chamomile Essential Oils^a^

	IC_50_ (μg/mL)			
sample	COX-1	COX-2	SI	5-LOX (% inh.)
*M. spicata*	21.17 ± 0.85	14.28 ± 0.93	0.67	92.1
*M. chamomilla*	39.05 ± 1.02	11.83 ± 0.87	0.30	94.6
NDGA	NT	NT		98.9 ± 1.1
celecoxib	8.2 ± 1.2 (μM)	0.73 ± 0.94 (μM)	0.08	NT
SC-560	9.80 ± 1.06 (nM)	4.40 ± 0.63 (μM)	>50	NT
Dup-697	9.5 ± 0.86 (μM)	70.44 ± 0.91 (nM)	0.007	NT

a NT: not tested.

On the other hand, LOX enzyme inhibitions were also evaluated due to the anti-inflammatory potential in the present work. While *M. spicata* essential oil inhibited the LOX enzyme by 92.1%, *M. chamomilla* essential oil showed 94.6% inhibition. In a previous study,^17^ another *M. spicata* essential oil sample was evaluated by our group for the LOX enzyme inhibition, where the results were comparable to the present study. The LOX enzyme inhibition was relatively higher, and this may be due to a higher carvone content of the essential oil compared to the previous study. Previous studies showed that carvone is a significant anti-inflammatory compound.^26^

### 2.4 Time-Dependent p38MAPK Enzyme Inhibition

**Figure 2:**
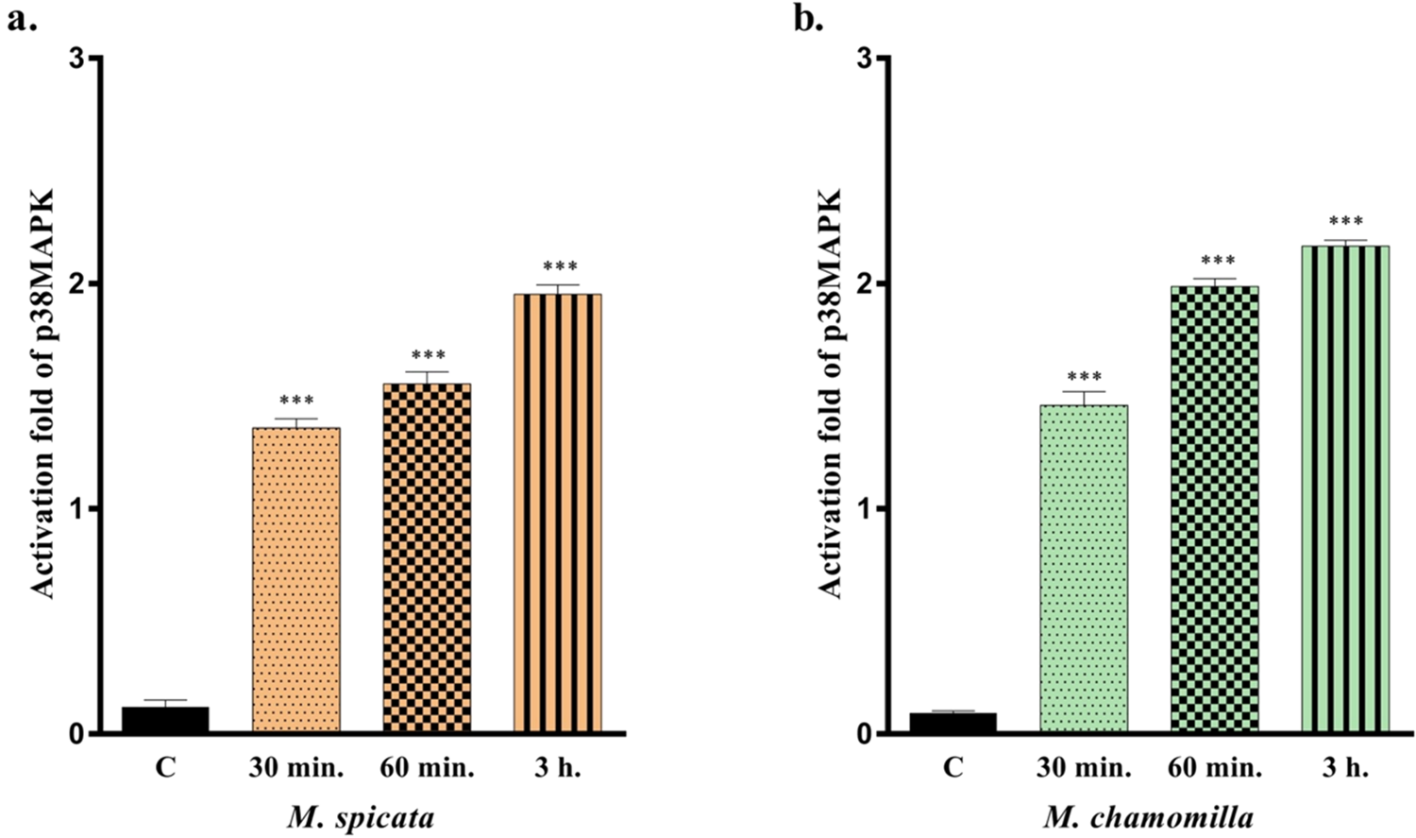
Effects of *M. spicata* (a) and *M. chamomilla* (b) essential oils on time-dependent p38MAPK enzyme inhibition. All data are statistically significant (mean ± SD, *n* = 3, ***P < 0.001, vertical bars indicate standard deviation values).

The apoptosis effects of *M. spicata* and *M. chamomilla* essential oils on the p38MAPK enzyme were evaluated in the present study. The experimental results showed that both essential oils induced p38 MAPK activities at different time intervals. As shown comparatively, the mean ± standard deviations are listed as illustrated in Figure 2, where the effects of essential oils on p38MAPK after 30 min, 60 min, and 3 h exposures were evaluated using an ELISA method. Compared to the control group, the exposure of *M. spicata* essential oil increased the enzyme activity by 1.2-, 1.5-, and 1.8-fold, where it was increased by 1.4-, 1.9-, and 2.1-fold for *M. chamomilla* oil. According to the recent literature, activation of the p38 MAPK pathway leads cells to apoptosis.^27^ When the caspase results and p38MAPK results in this study were evaluated, similar findings were obtained, which agrees with previous findings.

### 2.5 EGFR Analysis

In this present study, both essential oils were tested for the epidermal growth factor receptor (EGFR) inhibition potential, to the best of our knowledge for the first time. The experimental results showed that *M. spicata* and *M. chamomilla* inhibited the EGFR by 77.65 ± 1.48 and 85.97 ± 1.31%, respectively. Overexpression of the epidermal growth factor receptor plays a role in cancer development and growth. The interaction of EGFR with certain ligands is also associated with MAPK and plays a role in cancer development.^28^ Thus, EGFR inhibition is a targeted receptor in cancer therapy, as EGFR plays a role in cancer progression and development.

### 2.6 Caspase-3 Activity

**Figure 3:**
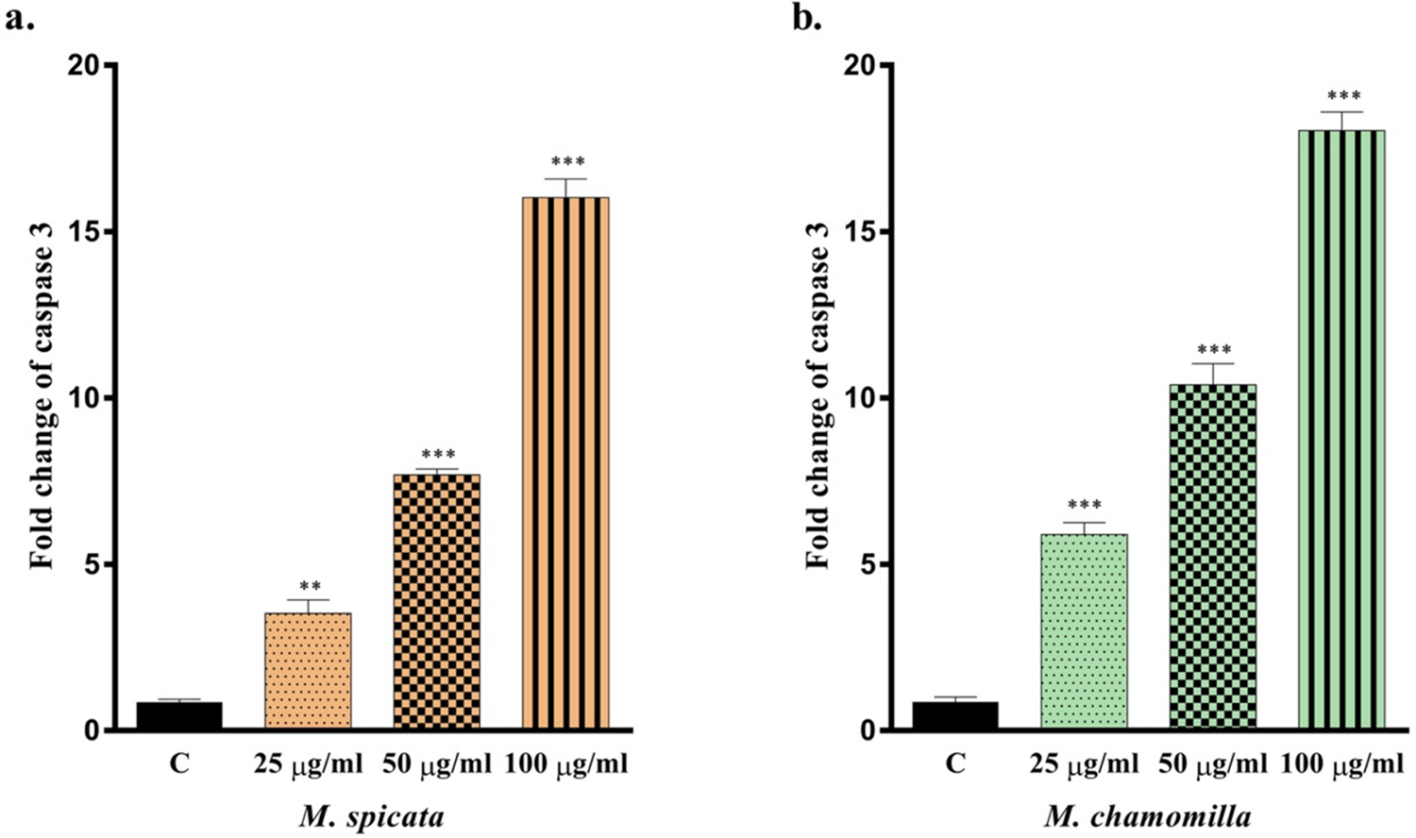
Effects of *M. spicata* (a) and *M. chamomilla* (b) essential oils on caspase-3. All data are statistically significant (mean ± SD, *n* = 3, **P < 0.01, ***P < 0.001, vertical bars indicate standard deviation values).

The effect of caspase-3, which is known to be one of the apoptotic cell death markers, was evaluated for the essential oil-induced cell death mechanism. U87MG cells treated at increasing essential oil concentrations were collected, where caspase-3 levels were examined using a commercial kit. The results showed an increase in caspase-3 levels, which was observed in all experimental groups. The effects of both essential oils at 25, 50, and 100 μg/mL concentrations were evaluated and compared. Depending on the concentration increase, the levels of caspase-3 increased as illustrated in Figure 3. In the groups treated with *M. spicata* oil, the caspase levels were increased by approximately 3-, 7-, and 15-fold, whereas in the groups treated with *M. chamomilla* essential oil, a 5-, 9-, and 17-fold increase was observed. Experimental results were compared using the Tukey test, where all results were statistically significant (Figure 3, *P < 0.05, **P < 0.01, ***P < 0.001, ****P < 0.0001 vertical bars indicate standard deviation values).

Overall, the experimentally tested *M. spicata* and *M. chamomilla* essential oils showed antiproliferative effects on selected cancer cells in the present study. The oils also showed significant COX and LOX enzyme inhibitions associated with inflammation and various cancers with a possible mode of action proposal. Furthermore, an outcome of the present study suggested that the oils may exert anticancer activity through activation of p38 MAPK signaling and activation of the apoptotic pathway.

In conclusion, considering the initial experimental results of this present study, it is worthwhile to investigate the *M. spicata* or *M. chamomilla* essential oils *in vivo* clinically for further anticancer drug discovery, especially in drug-resistant cancer cells and also in various combinations.

## 3 Materials and Methods

### 3.1 Chemicals and Reagents

Pharmacopoeia Grade Essential oils were acquired from Anoxymer International GmbH (Kümmersbruck, Germany) (*M. spicata* essential oil Batch Number: 20000369003, *M. chamomilla* essential oil Batch Number: 20004197007). Cell culture reagents were purchased from Gibco (San Diego, California). COX-1/COX-2 fluorometric inhibitory screening assay kit was acquired from Cayman Chemical Company (Ann Arbor, Michigan). p38MAPK enzyme and EGFR inhibition kit were obtained from Invitrogen (Carlsbad, California, ABD). Caspase-3 colorimetric assay kit and all of the other chemicals were purchased from Sigma-Aldrich.

### 3.2 GC-FID and GC/MS Analyses

The Agilent 6890N GC system was used. Simultaneous automatic injection was carried out using the same conditions in two identical columns [HP-Innowax FSC column (60 m × 0.25 mm, 0.25 μm film thickness, Agilent, Walt & Jennings Scientific, Wilmington, Delaware)] in the Agilent 5975 GC/MSD system. Relative percentages (%) of the compounds were calculated using the FID chromatograms. For idenfication and characterization in-house “Baser Library of Essential Oil Constituents” and various GC/MS Libraries such as MassFinder 3 Library, where authentic samples or the relative retention index (RRI) of *n*-alkanes were also considered,^29^ see, for details, Figures S1 and S2.

### 3.3 Cell Culture and Conditions

Human breast cancer cell lines MCF7 (ATCC HTB-22), human lung cancer cell lines A549 (ATCC CCL-185), human prostate cancer cell lines PC3 (ATCC CRL-1435), and human embryonic kidney cell lines HEK293 (ATCC CRL-1573) were from American Type Culture Collection and were cultured in Dulbecco’s modified Eagle’s medium/high glucose (DMEM, high glucose) or Dulbecco’s modified Eagle’s medium/F12 (DMEM, F12) supplemented with 10% heat-inactivated fetal bovine serum (FBS), 1% (v/v) antibiotic–antimycotics solution (100 U/mL penicillin,100 μg/mL streptomycin, and 0.25 μg/mL amphotericin B), and 1% (v/v) nonessential amino acids at 37 °C in a humidified 5% CO_2_ atmosphere. The cells were passaged every 3 days.^30^

### 3.4 Cytotoxicity Assay

Cytotoxic activities of *M. spicata* and *M. chamomilla* essential oils were determined by cell proliferation analysis using the standard 3-(4,5-dimethylthiazole-2 yl)-2,5-diphenyltetrazolium bromide (MTT) assay.^30^ Cells were cultured in 96-well plates of 10^5^ cells per well and 24 h prior to exposure. The essential oils were dissolved in DMSO (<1%) and diluted with medium for use in the MTT assay (0.1–2000 μg/mL). After incubation, the medium on the cells was removed and washed with D-PBS, and 30 μL of MTT was added from the stock solution (5 mg/mL, prepared in D-PBS). At the end of the 4 h incubation period, 150 μL of DMSO was added and mixed using a shaker at room temperature, followed by the measurement of the absorbances at a wavelength of 540 nm using a microplate reader (Spectramax i3, California). Colchicine was used as a positive control. Three independent experiments were performed at least, and results were reported as mean.

### 3.5 COX-1/COX-2 Enzyme Inhibitory Assay

The essential oils were tested using a commercial COX-1 (Ovine), COX-2 (human recombinant), fluorometric inhibitory screening assay kit, following the instructions recommended by the manufacturer (Cayman test kit 700100, Cayman Chemical Company, Ann Arbor, Michigan). 150 μL of assay buffer (0.1 M Tris-HCl pH 8.0), 10 μL of Heme, 10 μL of enzyme (COX-1 or COX-2), and 10 μL of the essential oil to be tested were added to the wells. After 5 min at room temperature, 10 μL of ADHP (10-acetyl-3 7-dihydroxy phenoxazine) and 10 μL of arachidonic acid were added to initiate the reaction. The plate was incubated at room temperature for 2 min, followed by fluorescence measured at 530 nm (excitation) and 585 nm (emission). Stock solution of the essential oils was prepared with below DMSO 1%. The triplicate experimental data were expressed as mean ± standard deviation (SD).^31^ For the percentage inhibitions (=%*I*), the formula below was used
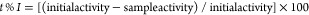


### 3.6 5-LOX Enzyme Inhibitory Assay

The assay reaction was started by adding linoleic acid solution, and the absorbance was observed at 234 nm for 10 min. The max concentration of essential oils tested was 20 μg/mL. The percent inhibition (%*I*) was calculated per minute in enzyme activity (without inhibitor) compared to the change in absorbance per minute of the test sample. Nordihydroguaiaretic acid (NDGA) was used as a positive control. Experiments were performed in triplicate, and the average results were reported as previously.^32^

### 3.7 p38MAPK Enzyme Inhibitory Assay

The ELISA Kit (Invitrogen) was applied in accordance with the manufacturer’s instructions.^33^ This assay was performed to detect p38 MAPK (α, β) in essential oil-treated cell lysates. 10^5^ cells/mL were collected and washed two times with cold PBS. The cells were dissolved in the cell extraction buffer [10 mM Tris, (pH 7.4), 100 mM NaCl, 1 mM EDTA, 1 mM EGTA, 1 mM NaF, 20 mM Na_4_P_2_O_7_, 2 mM Na_3_VO_4_, 1% Triton X-100, 10% glycerol, 0.1% SDS, and 0.5% deoxycholate] for 30 min on ice, vortexed at 10 min intervals, and centrifuged at 13 000 rpm for 10 min at 4 °C. Standard, control and samples were put into the striped wells and incubated at room temperature for 2 h. The p38 MAPK detection antibody solution was added into the wells and incubated at different times (30, 60, and 90 min), followed by the addition of antirabbit IgG HRP solution and stabilized chromogen to the wells at the appropriate temperature and incubation times for blue well coloration. Finally, a stop solution was added to each well, and the change of solution from blue to yellow was monitored to measure the absorbance at 450 nm.^34^

### 3.8 EGFR Inhibitory Assay

The kinase assay protocol was tested according to the manufacturer’s instructions and modified from the literature.^35^ The EGFR and substrate were diluted using the assay buffer and ATP solution. 2 μL of the essential oil at different concentrations (dissolved in max 1% DMSO) was placed in each well. 4 μL of kinase working stock and 4 μL of ATP substrate working stock were added and incubated for 1 h at room temperature. After the incubation period, the concentration-dependent kinase inhibition effects of the essential oils were measured using the ADP-Glo kinase assay using the luminescence plate reader device (SpectraMax i3, California).

### 3.9 Caspase-3 Activity Assay

The 96-well plate colorimetric “Caspase-3 Assay Kit” (Sigma, CASPC3), following the manufacturer’s instructions, was used.^33^ Cells were treated with cell lysis buffer, incubated on ice for 10 min, and centrifuged at 10 000*g* for 5 min at 4 °C. The reaction mixture (total volume, 100 μL) containing 30 μL of cell lysate and 10 μL of the caspase-3 substrate acetyl-Asp-Glu-Val-Asp-p-nitroanilide (final concentration, 200 μM) in assay buffer was prepared. The control group reaction mix contained 30 μL of cell lysate and 10 μL (final concentration, 20 μM) of the specific caspase-3 inhibitor acetyl-DEVD-CHO in assay buffer. Both mixtures were incubated at 37 °C for 90 min and absorbance was read at 405 nm.^33^ Fold change of caspase-3 the essential oils was calculated by comparison with the control group.

### 3.10 Statistical Analysis

Statistical analysis was observed by using GraphPad Prism 8 (GraphPad Software, Inc., San Diego, California; Version 8.4.3). The statistical evaluation was performed with one-way analysis of variance (ANOVA) followed by Dunnett’s or Tukey post hoc tests to multiple comparisons. The limit of significance was accepted as *P* < 0.05. All repeated experiments were in triplicate.

## Supporting Information Available

GC-MS chromatograms of the essential oils (PDF)

## Notes

This article does not contain any studies with human participants or animals performed by any of the authors.
