# Supplementary material for: In Vitro Anti-Inflammatory and Anticancer Evaluation of Mentha spicata L. and Matricaria chamomilla L. Essential Oils
Source: ACS Omega. 2023 May 6;8(19):17143–50. doi: 10.1021/acsomega.3c01501 (PMC10193545; doi:10.1021/acsomega.3c01501)
Supplement: Supplementary file 1 — ao3c01501_si_001.pdf [file ao3c01501_si_001.pdf]

In vitro anti-inflammatory and anticancer evaluation of _Mentha spicata_ L. and _Matricaria chamomilla_ L. essential oils

Sevde Nur Biltekin[1,2], Ayşe Esra Karadağ[3], Fatih Demirci[4,5], Betül Demirci[4]*

[1]Department of Pharmaceutical Microbiology, School of Pharmacy, Istanbul Medipol University, Istanbul, Türkiye.

[2]Department of Molecular Biology and Genetics, Institute of Graduate Studies in Sciences, Istanbul University, Istanbul, Türkiye

[3]Department of Pharmacognosy, İstanbul Medipol University, Faculty of Pharmacy, 34810, İstanbul, Türkiye

[4]Department of Pharmacognosy, Faculty of Pharmacy, Anadolu University, 26470-Eskişehir, Türkiye

[5]Department of Pharmacognosy, Faculty of Pharmacy, Eastern Mediterranean University, 99450-Famagusta, N.Cyprus, Türkiye

[∞] This work is dedicated to those who passed away during the devastating earthquakes of the century in Anatolia, Feb 2023.

[*]Corresponding author: Betül Demirci, Department of Pharmacognosy, Faculty of Pharmacy, Anadolu University, 26470-Eskişehir, Türkiye; e-mail: bdemirca@anadolu.edu.tr

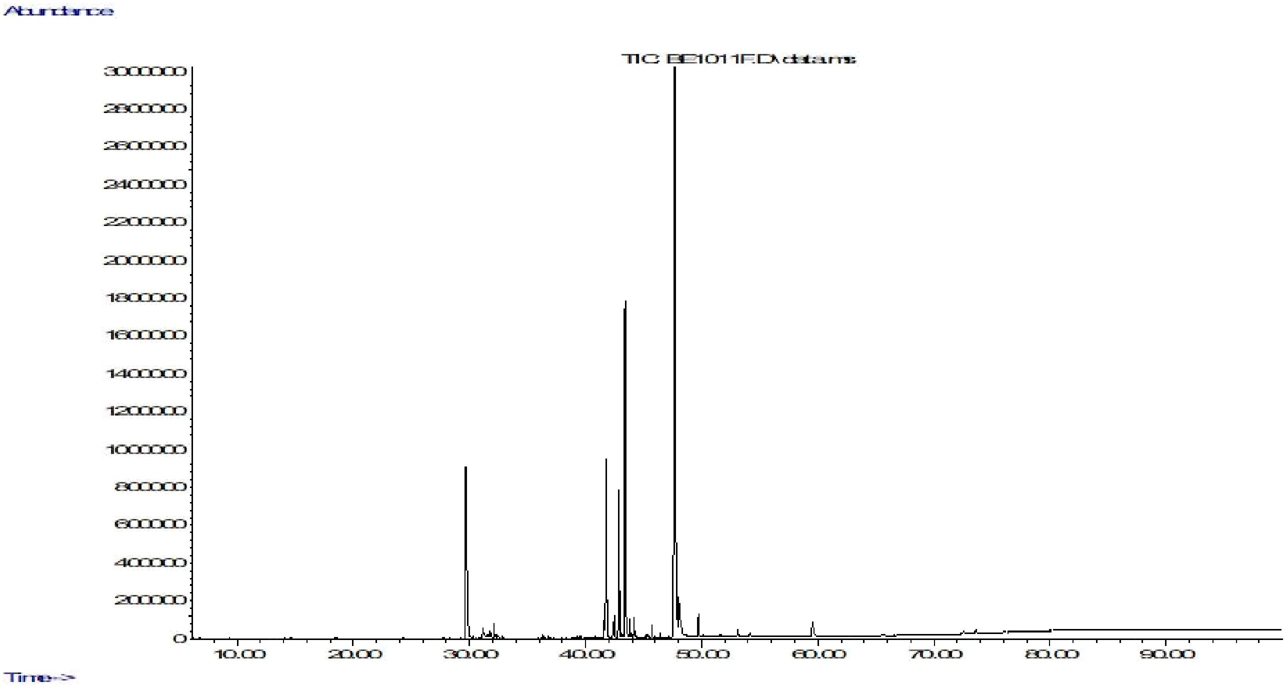

Figure S1. GC/MS Chromatogram of *M. chamomilla* essential oil

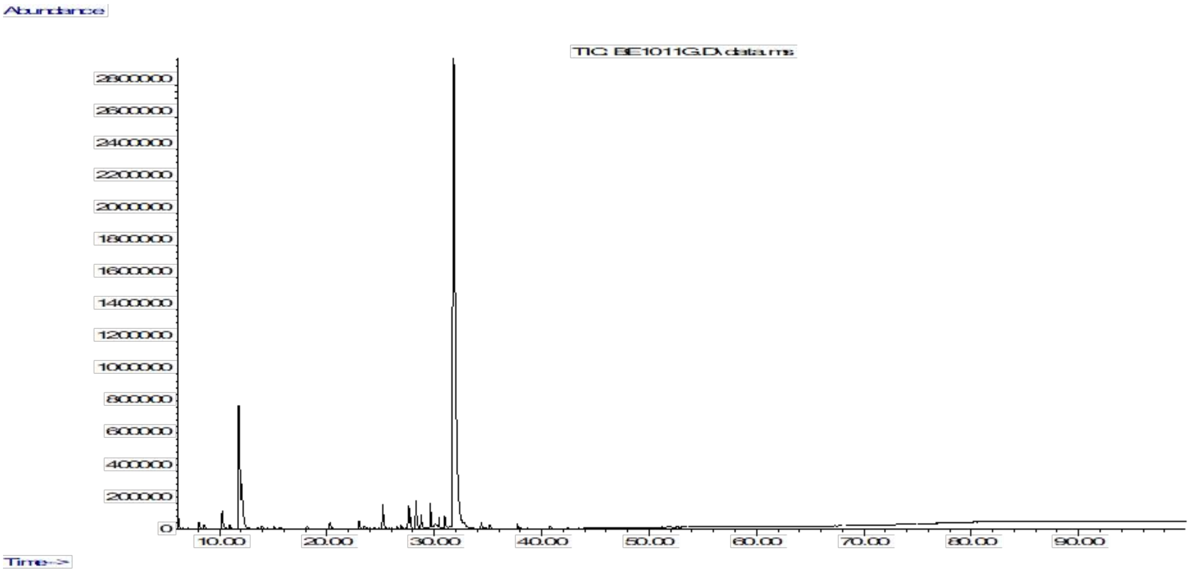

Figure S2. GC/MS Chromatogram of *M. spicata* essential oil
